# Folate-Targeted Nanoliposomal Chemophototherapy

**DOI:** 10.3390/pharmaceutics15102385

**Published:** 2023-09-26

**Authors:** Upendra Chitgupi, Yiru Qin, Sanjana Ghosh, Breandan Quinn, Kevin Carter, Xuedan He, Ulas Sunar, Jonathan F. Lovell

**Affiliations:** 1 Department of Biomedical Engineering, University at Buffalo, The State University of New York, Buffalo, NY 14260, USA; upendrac@buffalo.edu (U.C.); yqin@gdoh.org (Y.Q.); sghosh22@buffalo.edu (S.G.); breandan@buffalo.edu (B.Q.); kcarter@buffalo.edu (K.C.); xuedanhe@buffalo.edu (X.H.); 2 Department of Biomedical Engineering, Stony Brook University, Stony Brook, NY 11794, USA; ulas.sunar@stonybrook.edu

**Keywords:** liposome, folate, folic acid, chemophototherapy, photodynamic therapy, doxorubicin

## Abstract

Light-responsive liposomes have been developed for the on-demand release of drugs. However, efficient delivery of chemotherapeutic drugs to tumor for cancer theranostics remains a challenge. Herein, folic acid (FA), an established ligand for targeted drug delivery, was used to decorate light-sensitive porphyrin-phospholipid (PoP) liposomes, which were assessed for FA-targeted chemophototherapy (CPT). PoP liposomes and FA-conjugated PoP liposomes were loaded with Doxorubicin (Dox), and physical properties were characterized. In vitro, FA-PoP liposomes that were incubated with FA receptor-overexpressing human KB cancer cells showed increased uptake compared to non-targeted PoP liposomes. Dox and PoP contributed towards chemophototherapy (CPT) in vitro, and PoP and FA-PoP liposomes induced cell killing. In vivo, mice bearing subcutaneous KB tumors treated with PoP or FA-PoP liposomes loaded with Dox, followed by 665 nm laser treatment, had delayed tumor growth and improved survival. Dox delivery to tumors increased following laser irradiation for both PoP and FA-PoP liposomes. Thus, while Dox-FA-PoP liposomes were effective following systemic administration and local light irradiation in this tumor model, the FA targeting moiety did not appear essential for anti-tumor responses.

## 1. Introduction

Gynecological cancer is a major cause of death in women worldwide [1,2,3]. Ovarian cancers have been reported to overexpress folate-binding proteins at various levels [4,5]. The alpha isoform of the folic acid (FA) receptor has been used as a target for selective drug delivery via FA [5]. The general basis for targeted drug delivery is that cytotoxic chemotherapy drugs lack tumor specificity, resulting in accumulation of the drug in healthy tissue leading to unwanted side effects [6,7]. Nanocarriers like micelles, liposomes, and other nanoparticles have been explored for drug delivery [7,8]. Novel methods have been developed for the delivery of nanoparticles to achieve improved therapeutic efficacy [9,10,11]. Liposomes have shown promise for cancer treatment in the clinic [12]. In preclinical research, ligand-decorated liposomes have demonstrated potential in active targeting applications for cancer therapeutics. Active targeting makes use of ligand-receptor binding affinity for improved drug delivery and therapy. Strong interaction between the ligand on the surface of the nanoparticle and the receptor on the tumor cells could result in improved binding and thus enhance the delivery of the drug. FA possesses high binding affinity to FA receptors (Kd∼10^−10^ M) and therefore has been explored as a target in cancer therapeutics [13]. The FA receptor alpha is a glycosylphosphatidylinositol anchored receptor on the cell surface that has been established as a tumor marker for certain cancers [14,15]. FA receptors bind to FA followed by endocytosis, finally resulting in the release of FA at acidic pH in endosomes [16,17]. Active targeting can make use of the FA receptor high affinity for the FA ligand, resulting in uptake of FA-modified nanocarrier encapsulated with chemotherapeutic drugs.

Photodynamic therapy (PDT) is a local cancer treatment which uses the therapeutic potential of light for treatment. PDT involves the use of light to activate a photosensitizer, which then releases reactive oxygen species to destroy the irradiated tissue [18]. PDT has the potential to enhance drug delivery by altering the permeability of the tumor vasculature. Researchers have also explored treating certain types of cancers with poor prognosis, for example, ovarian cancer, with PDT [19,20]. PDT is generally minimally invasive and provides high specificity as the light can be limited to a target volume of interest. Additionally, PDT agents have optical imaging capabilities. Imaging-assisted PDT treatment can provide real-time feedback on drug accumulation and the degree of tissue damage, thereby assisting in optimizing the treatment [21,22]. PDT applications in cancer therapy have utilized FA receptors for targeted therapy [3,23]. One targeting strategy involves the synthesis of FA-photosensitizer conjugates for FA receptor-mediated PDT [24]. To improve the specificity and drug accumulation approaches like photoimmunotherapy, which involves the delivery of PS through conjugation to an antibody, have also been explored as a therapeutic for cancer [25]. In this study, we demonstrate the use of a combination approach comprising both chemotherapy and phototherapy, referred to as chemophototherapy (CPT). Combination therapies such as CPT have been demonstrated to be more effective than stand-alone treatments [26,27,28,29]. Small molecules like anthracycline drugs that serve as chemotherapeutics have been routinely used in cancer treatments. In this study, liposomes were loaded with Dox which is a well-known chemotherapeutic drug. To enhance the targeting of the drug, Dox was loaded into sterically stabilized liposomes, which have increased circulation time following administration. Additionally, a small amount of PoP, a porphyrin-based light-sensitive lipid, was incorporated into the liposomes as a liposomal photosensitizer [30]. Our group has demonstrated that the incorporation of PoP in liposomes is compatible with cancer drug loading and confers light-triggered drug release [31]. PoP liposomes have been previously shown to release the encapsulated drugs when irradiated with 665 nm light [32]. In this study, we investigate Dox encapsulated in FA-PoP liposomes for targeting FA receptor overexpressing cancer cells, which can then be irradiated with 665 nm light for CPT.

## 2. Materials and Methods

Materials: 1,2-distearoyl-sn-glycero-3-phosphocholine (DSPC, #LP-R4-076) and 2-distearoyl-sn-glycero-3-phosphoethanolamine-N-[methoxy(polyethylene glycol)-2000] (DSPE-PEG-2K, LP-R4-039) were purchased from Corden Pharma. Cholesterol (Wilshire Technologies, #57-88-5) and DSPE-PEG-2k-Folic-Acid (DSPE-PEG-FA, Avanti# 880124) were procured. PoP was synthesized as reported [33].

Liposome preparation: PoP liposomes were prepared with [DSPC:Cholesterol:DSPE-PEG-2K:PoP] in the molar ratio [53:40:5:2]. Folic acid (FA) PoP liposomes were prepared by modifying PoP liposome composition to [DSPC:Cholesterol:PEG:DSPE-PEG-2K:DSPE-PEG-FA:PoP] with the ratio [53:40:4.7:0.3:2]. Liposomes were prepared by ethanol injection method. In total, 100 mg of total lipids (57.1 mg of DSPC, 21.1 mg of cholesterol, 19.1 mg of PEG-2000, 2.8 mg of PoP) were dissolved in 1 mL of ethanol and heated to 65 °C using a heating block. Then, 4 mL of 250 mM ammonium sulfate solution (pH 5.5) heated to 65 °C were added to lipids dissolved in ethanol. Liposomes were passed through a LIPEX nitrogen pressurized extruder (Northern Lipids) 10 times (through 200, 100, and 80 nm filters). FA liposomes were prepared using the same method with the aforementioned liposome composition. Liposomes were then dialyzed against a 10 mM histidine buffer (pH 6.5) with 10% sucrose to remove ethanol and external ammonium sulfate. Liposomes were dialyzed for 4 cycles of 4 h each.

### 2.1. Characterization

Liposomes prepared by the extrusion method were first characterized through size, polydispersity index, and zeta potential. Dynamic light scattering and zeta potential studies were carried out using Malvern ZS90. The fluorescence of PoP liposomes was analyzed using a Photon Technology International (PTI) fluorimeter. Self-quenching of doxorubicin-loaded liposomes was measured using a TECAN plate reader.

### 2.2. Doxorubicin Encapsulation and Release

Dialyzed liposomes were loaded with Dox using the remote loading mechanism with ammonium sulfate gradient [34]. Dox was incubated with dialyzed liposomes at 1:6 drug to lipid ratio for 1 h at 60 °C. Encapsulation of Dox in liposomes was measured by running the Dox encapsulated liposomes over a G-75 Sephadex column. In total, 100 µL of liposomes were diluted to 500 µL and added to 10 mL G75 Sephadex column. Twenty-four fractions of 0.5 mL each were collected. The fluorescence of the samples in the fractions was measured using a TECAN fluorescence microplate reader (excitation 480 nm and emission 590 nm). For fluorescence measurement, 30 µL of collected liposome fraction were added to 170 µL of PBS and 2 µL of 10% triton-x 100. The loading efficiency of the drug was estimated using the sum of fluorescence values of fractions 3–11 and comparing it to the total fluorescence over all collected fractions. For serum stability studies, Dox liposomes were incubated in 50% Fetal Bovine Serum (FBS) at 37 °C. To measure the Dox leakage, 200 µL aliquots were added to a 96-well plate and measured using a TECAN fluorescence microplate reader. Drug release from Dox-loaded liposomes was monitored in real-time with the help of a Photon Technology International fluorimeter. Then, 10 mg mL^−1^ of Dox-PoP and Dox-FA-PoP liposomes were added to 50% FBS prewarmed to 37 °C to a final concentration of 50 µg mL^−1^. Dox-encapsulated liposomes were irradiated with a power tunable 665 nm laser diode (RPMC Lasers) for 15 min. Dox release from liposomes with no laser treatment was monitored using a similar setup. For all release studies, Dox fluorescence was read at 480 nm excitation and 590 nm emission.

### 2.3. In Vitro Studies

In vitro cell-based studies were carried out on human lung carcinoma (A549) and KB cell lines. Cells were obtained from the American Type and Culture Collection. A549 cells were cultured in DMEM supplemented with 10% FBS and antibiotics. KB cells were cultured in FA-free RPMI 1640 supplemented with 10% FBS and antibiotics. Cells were subcultured into 25 cm^3^ flasks and always maintained in a 5% CO_2_ incubator at 37 °C. For in vitro experiments relating to cytotoxicity, chemophototherapy, and binding studies, 1 × 10^4^ cells per well were seeded on a 96-well plate.

For cell binding studies using flow cytometry, 1 × 10^5^ KB cells (cell density per well) grown in a 24-well plate were incubated with varying concentrations of PoP and FA-PoP empty liposomes for 45 min. KB cells treated with liposomes were washed with PBS thrice after incubation. To detach the cells, 0.4 mL of trypsin were added per well and incubated at 37 °C for 5 min. RPMI media (0.5 mL) was added, and samples were centrifuged. Centrifuged samples were suspended in 0.1% BSA, and flow cytometry studies were performed on BD LSRFortessa X-20 flow cytometer. The supernatant was discarded, and cells were washed with PBS twice. For FA receptor blocking studies, cells were preincubated with a 100-fold molar excess of FA for 1 h before treating the cells with PoP or FA-PoP liposomes.

For binding studies, KB or A549 cells were incubated with PoP or FA-PoP liposomes for 45 min unless otherwise noted. Cells were washed with PBS thrice post-incubation, and 200 µL of PBS per well were added to 2 µL of 10% triton-X 100 to lyse the cells. After 5 min, the PoP fluorescence of each well was measured using a TECAN fluorescence microplate reader. Fluorescence of PoP liposomes incubated with cells was compared with FA-PoP liposome wells incubated cells. For imaging studies, KB cells were incubated with PoP or FA-PoP liposomes for 45 min and washed with PBS thrice. Cells were imaged using an EVOS FL microscope using the PoP (ex:420 nm/em:665 nm) channel.

For PDT studies, KB cells were seeded in a 96-well plate at 1 × 10^4^ per well, and the plate was incubated at 37 °C for 24 h. Dox-loaded or empty liposomes were added to each well at the indicated concentration and incubated for 45 min. Post incubation, cells were washed with PBS twice and fresh media with FBS was added. Further, 96-well plates incubated with liposomes were irradiated with a 665 nm laser at 39 mW cm^−2^. Irradiation time was adjusted to result in a fluence between 0 and 20 J cm^−2^.

Phototoxicity of PoP and FA-PoP liposomes was assessed with XTT cell viability assay. In total, 50 μg mL^−1^ of XTT (2,3-Bis(2-Methoxy-4-Nitro-5-Sulfophenyl)-2H-Tetrazolium-5-Carboxanilide) and 30 μg mL^−1^ of PMS (N-methyl dibenzopyrazine methyl sulfate) in PBS were added to each well. Approximately 3 h later, the 96-well plate was read at 450 nm with 630 nm as a reference. Cell viability was calculated based on the control cells, which remained untreated throughout the experiment. Error bar indicates standard deviation from the mean for *n* = 3 or 4.

### 2.4. Tumor Biodistribution

All animal experiments were performed in accordance with policies approved by the University at Buffalo Institutional Animal Care and Use Committee. Athymic nude mice were subcutaneously inoculated with 2 × 10^6^ KB cells on both flanks. When the tumor size reached 6–8 mm, mice were randomly grouped into 4 cohorts of 4 mice and were injected with either Dox-PoP (2 cohorts) or Dox-FA-PoP (2 cohorts) at 4 mg kg^−1^. Post injection, the tumor on the right flank received laser treatment (665 nm) at 150 mW cm^−2^ for 27.7 min, resulting in 150 J cm^−2^. Tumors were harvested after 4 h and 24 h and homogenized with nuclear lysis buffer (250 mM sucrose, 5 mM Tris-HCl, 1 mM MgSO_4_, 1 mM CaCl_2_, pH 7.6). Homogenates were then extracted overnight with 0.075N HCI and 90% isopropanol at −20 °C. The following day, samples were centrifuged at 5000× *g*, and the fluorescence of the samples was measured at 480/590 nm for Dox and concentrations were determined based on a standard curve.

### 2.5. Tumor Inhibition and Studies

Athymic female nude mice were subcutaneously inoculated with 2 × 10^6^ KB on the right flank. When tumor volumes reached 4–6 mm in diameter, mice were grouped into cohorts of 5–6 and injected with (1) Saline, (2) FA-PoP + laser, (3) Dox-PoP + laser, (4) Dox-FA-PoP, and (5) Dox-FA-PoP + laser. A Dox dose of 3 mg kg^−1^ was injected. Mice cohorts requiring laser treatment were treated with laser (150 mW cm^−2^ for 44.4 min, 400 J cm^−2^) 1 h after injection. Tumor temperature was monitored during laser treatment using a FLIR temperature gun. Tumor volume was calculated using the ellipsoid formula. The body weights of the mice were monitored for 4 weeks post-treatment. Mice were sacrificed when the tumors reached 1.0 cm.

### 2.6. Ex Vivo Fluorescence Imaging

Tumors were harvested from mice sacrificed 4 h and 24 h post-injection. A part of the tissue was immediately immersed in OCT and SNAP frozen in liquid nitrogen. Frozen sections were prepared using a microtome-cryostat (OTF5000, Bright Instruments). Slides prepared with cryo-stat were immediately imaged with an EVOS FL microscope. Dox fluorescence was imaged at exc./em. 480 nm/590 nm and PoP fluorescence exc./em. at 420/665 nm.

### 2.7. In Vivo Fluorescence Imaging

Nude mice were inoculated intraperitoneally with 5 × 10^6^ human ovarian cancer (A2780) cells. After 21 days, mice were injected with 100 nM PoP containing FA-PoP liposomes. Mice were sacrificed after 48 h, and whole-body imaging was performed using IVIS Lumina.

### 2.8. Statistical Analysis

For cell viability studies, one-way ANOVA was performed on the results, followed by post hoc Tukey’s HSD test. * indicates *p* < 0.05, ** indicates *p* < 0.01, and *** indicates *p* < 0.005. For in vivo dox in tumor results, an unpaired *t*-test was performed.

## 3. Results

### 3.1. Preparation and Characterization of Liposomes

Liposomes used in these studies were prepared with the following formulation: 53 molar % DSPC, 40 molar % Cholesterol, 5 molar % DSPE-PEG-2k, and 2 molar % PoP. Folic-acid containing PoP (FA-PoP) liposomes were formed with the following formulation: 53 molar % DSPC, 40 molar % Cholesterol, 4.7 molar % DSPE-PEG-2k, 2 molar % PoP, and 0.3 molar % DSPE-PEG-2k-FA. The increase in DSPE-PEG-2k-FA was compensated by a reduction in DSPE-PEG-2k. Furthermore, 5% DSPE-PEG-2k was selected since it was established for drug loading and light-triggered drug release [31]. Doxorubicin was loaded in liposomes using the remote loading method and an ammonium sulfate gradient [34]. Unless otherwise noted, FA-PoP liposomes were prepared with 0.3% DSPE-PEG-2k-FA for all studies. Previous studies have shown 2% molar PoP as optimal for drug release; therefore, 2% molar PoP was incorporated into the current formulation [33]. Liposomes were extruded, and the size and polydispersity index (PDI) of empty liposomes and Dox-loaded PoP liposomes were measured. As shown in Table 1, the average size of empty PoP and FA-PoP liposomes was around 100 nm and were fairly monodisperse with PDI between 0.05 and 0.1. Dox-loaded PoP liposomes were around 100 nm whereas Dox-loaded FA-PoP liposomes were marginally larger in size at an average diameter of 117 nm. PDI of Dox-loaded liposomes was less than 0.12. We did not assess morphology of liposomes in this study, but prior research showed that Dox forms fibrous aggregates in the aqueous core of PoP liposomes, as expected for actively loaded Dox liposomes [32].

**Figure 1:**
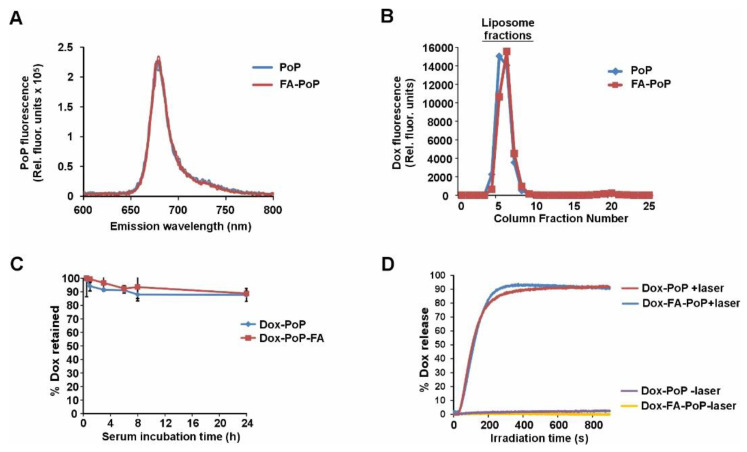
Drug loading and triggered release of Dox from PoP and FA-PoP liposomes. (**A**) Fluorescence emission spectra of PoP in PoP or FA-PoP liposomes. (**B**) Dox fluorescence from liposome fractions collected from gel filtration (G75 Sephadex) column. (**C**) Serum stability of Dox-loaded PoP or FA-PoP liposomes incubated in 50% FBS. (**D**) Light-triggered release of Dox from liposomes incubated in 50% FBS. Laser irradiation was provided by a 665 nm laser diode.

To demonstrate that DSPE-PEG-FA was incorporated into the lipid bilayer without altering the photophysical properties of PoP, the fluorescence emission spectra of PoP and FA-PoP liposomes were evaluated. Both PoP and FA-PoP liposomes exhibited similar spectra with maxima around 665 nm, as shown in Figure 1A. To assess the drug-loading efficiency of PoP and FA-PoP liposomes, the liposomes were loaded with Dox. Dox-loaded liposomes were subjected to gel filtration to separate them via size exclusion chromatography. Column fractions showed Dox co-eluted with the liposomes between fractions 3 and 11 for both Dox-PoP and Dox-FA-PoP liposomes (Figure 1B). Free doxorubicin eluted in later fractions was identified by a small peak around fraction 20. Based on fluorometric analysis of Dox in the collected fractions, drug loading was found to be over 99% for both Dox-PoP and Dox-FA-PoP liposomes. To assess the stability of liposomes in physiological fluids in vitro, Dox-PoP and Dox-FA-PoP liposomes were incubated in 50% fetal bovine serum (FBS) at 37 °C for 24 h. Drug leakage was found to be approximately 10% from the liposomes after 24 h incubation at 37 °C (Figure 1C). This reflects a stable drug-loaded liposome formulation. To measure the rate of light-triggered drug release, Dox-PoP and Dox-FA-PoP liposomes diluted in FBS were irradiated with 665 nm light at 200 mW cm^−2^. Drug release of Dox was monitored using real-time fluorometric analysis. As shown in Figure 1D, incorporating DSPE-PEG-2k-FA did not impact the release rate or the release profile of Dox from liposomes. In total, 50% of the drug (T_50%_) was released from the liposomes within 2 min when irradiated with laser. Without laser exposure (Figure 1D), there was either no release or negligible Dox release (<2%) in the same time frame. We previously reported that the mechanism of release of Dox from PoP liposomes under irradiation is PoP-mediated photo-oxidation of lipids and cholesterol in the bilayer, leading to membrane permeabilization [35].

### 3.2. Cellular Binding and Uptake

**Figure 2:**
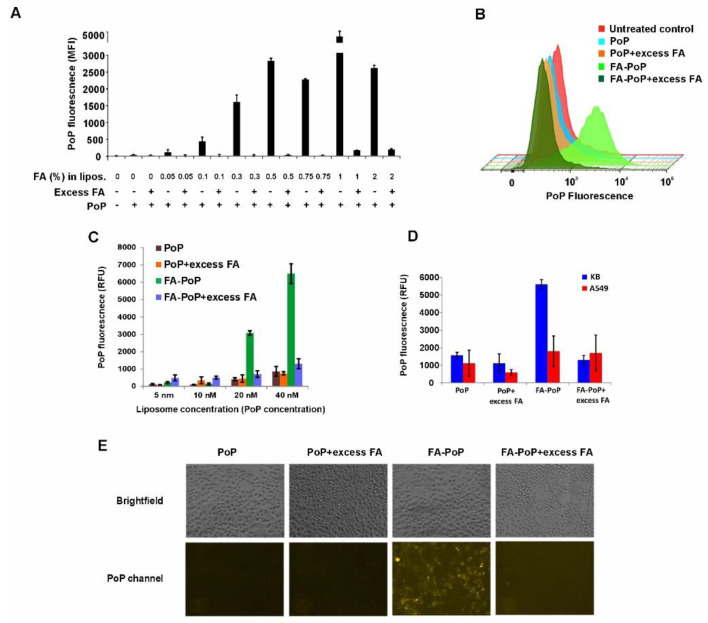
Cellular binding and uptake of FA-PoP liposomes. (**A**) PoP mean fluorescence intensity (MFI) of PoP and FA-PoP liposomes incubated with KB cells and measured with flow cytometry. Liposomes were prepared with varying percentages of FA, as indicated. (**B**) Representative PoP uptake histogram of PoP and FA-PoP samples incubated with KB cells. (**C**) Cellular uptake of liposomes measured by fluorometric analysis of cell lysate. Cells were incubated with increasing concentrations of PoP or FA-PoP liposomes at the indicated PoP concentration. (**D**) Uptake of liposomes (40 nM PoP concentration) in folate-receptor positive KB cells and folate-receptor negative A549 cells incubated for 45 min. (**E**) Microscopy images of KB cells incubated with indicated liposomes (40 nM PoP basis) for 45 min. PoP was read at 420/665 exc./em. For folate-blocking studies (indicated with excess FA in the figure legend), 500–1000 fold excess FA was used.

To assess the cellular targeting of FA-decorated liposomes, FA-PoP liposomes or PoP liposomes were incubated in vitro with KB cells, which are known to overexpress folate-receptor alpha [36]. PoP and FA-PoP liposomes were incubated with KB cells in folate-free media for 45 min. Cells were washed, and PoP uptake was analyzed using flow cytometry. Non-targeted PoP liposomes showed minimal fluorescence, whereas KB cells incubated with FA-PoP showed concentration-dependent uptake patterns, as shown in Figure 2A. Minimal PoP fluorescence observed in cells incubated with PoP liposomes would be expected from a small amount of nonspecific binding and uptake via endocytosis. Increasing PoP fluorescence trend with higher folic acid on the liposomes confirmed folic acid-dependent uptake of liposomes in KB cells. Additionally, cells preincubated with 500-fold of excess free FA showed a decrease in uptake, further confirming folate-specific uptake. Although there was increased binding and uptake of liposomes that incorporated higher FA amounts, liposomes made with FA higher than 0.3% tended to aggregate in storage. Previous studies have used targeting of liposomes with 0.3% FA, and since this flow cytometry results showed the 0.3% formulation had strong FA-dependent targeting, we prepared liposomal formulation with 0.3% DSPE-PEG2K-FA for further studies [37]. Figure 2B shows a representative histogram of fluorescence originating from PoP and 0.3% FA-PoP liposomes incubated with KB cells with/without the addition of excess FA. Cells incubated with FA-PoP liposomes showed PoP fluorescence orders of magnitude higher than non-targeting PoP liposomes or untreated control cell samples.

To determine if FA receptor-binding was dose-dependent, PoP and FA-PoP liposomes at different concentrations were incubated with KB cells in folate-free media for 45 min. Cells were washed with PBS and lysed using 1% Triton X-100. Fluorescence of the cell lysate was measured using a microplate reader. PoP fluorescence was 5–6-fold higher for FA-PoP treated cells compared to cells treated with non-targeting PoP liposomes (Figure 2C). For higher incubation concentrations, FA-PoP liposomes showed increasing PoP fluorescence, thereby signifying dose-dependent uptake of FA-PoP liposomes. To further validate FA receptor-dependent uptake of FA-PoP liposomes, PoP and FA-PoP liposomes were added to KB (folate receptor positive) and A549 (folate receptor negative) cells independently. KB cells incubated with FA-PoP liposomes showed 2.5-fold higher uptake compared to A549 cells. Excess FA blocking decreased the uptake of FA-PoP liposomes for KB cells, whereas A549 cells did not show a significant change in fluorescence with blocking (Figure 2D). FA-PoP and PoP liposomes were incubated with KB cells for 45 min, and cells were then imaged with fluorescence microscopy (Figure 2E). Cells incubated with FA-PoP liposomes showed visibly higher fluorescence, whereas the PoP liposome sample exhibited lower fluorescence. Additionally, KB cells preincubated with excess FA in vitro to block FA receptor binding showed decreased PoP fluorescence. These in vitro results together establish that FA receptor-mediated binding and uptake of FA-PoP liposomes occurred.

### 3.3. Chemophototherapy

**Figure 3:**
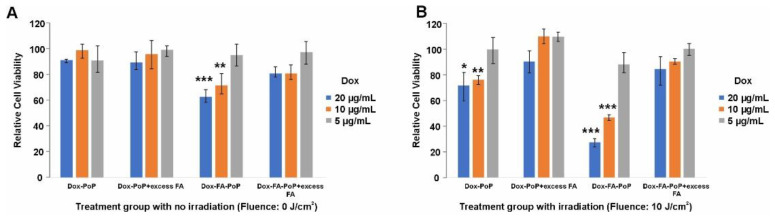
In vitro chemophototherapy of Dox-FA-PoP liposomes. (**A**) Dark toxicity of Dox-PoP and Dox-FA-PoP liposomes at indicated concentrations incubated with KB cells for 45 min. (**B**) Cell viability of cells incubated with liposomes followed by laser irradiation at 10 J cm^−2^. Laser irradiation studies were carried out using a 665 nm laser. Statistical difference compared to control cells with * indicating *p* < 0.05, ** indicating *p* < 0.01, and *** indicating *p* < 0.005.

To evaluate the phototherapeutic properties of Dox-PoP and Dox-FA-PoP liposomes, Dox-loaded PoP and FA-PoP liposomes were incubated with KB cells for 45 min. With no laser irradiation, KB cells remained healthy and displayed no signs of dark toxicity for Dox-loaded PoP liposomes. KB cells incubated with Dox-loaded FA-PoP liposomes exhibited dark toxicity at 20 and 10 µg mL^−1^ Dox, leading to a reduction of ~30% and ~20% cell viability, respectively (Figure 3A). Cell viability for KB cells incubated with Dox-FA-PoP at 20 and 10 µg mL^−1^ was statistically significant. Dark toxicity at higher concentrations can be attributed to the increased binding and uptake of the chemotherapeutic loaded in FA-decorated PoP liposomes. On the other hand, KB cells preincubated with excess free FA did not show a reduction in cell viability, indicating folate receptor-mediated uptake of Dox-FA-PoP liposomes. Excess FA blocking results indicate the uptake mechanism for these liposomes to be via FA receptor binding. Cells incubated with Dox-PoP and Dox-FA-PoP liposomes followed by irradiation with 665 nm laser at 10 J cm^−2^ showed a significantly higher loss in cell viability compared to incubation without laser irradiation (chemotherapy alone), as shown in Figure 3B. Dox-PoP liposomes incubated at higher concentrations with subsequent laser irradiation showed significant cell death. However, Dox-FA-PoP showed a significant decrease in cell viability with *p*-values < 0.005 at higher incubation concentrations. As expected, samples preincubated with excess FA showed minimal cell death post-laser treatment. Similar trends can be observed in Dox-PoP and Dox-FA-PoP, where chemophototherapy exhibited increased cell death compared to chemotherapy alone. However, Dox-FA-PoP treated samples generally resulted in more significant cell death in comparison with Dox-PoP, irrespective of chemotherapy or chemophototherapy.

### 3.4. Tumor Inhibition

**Figure 4:**
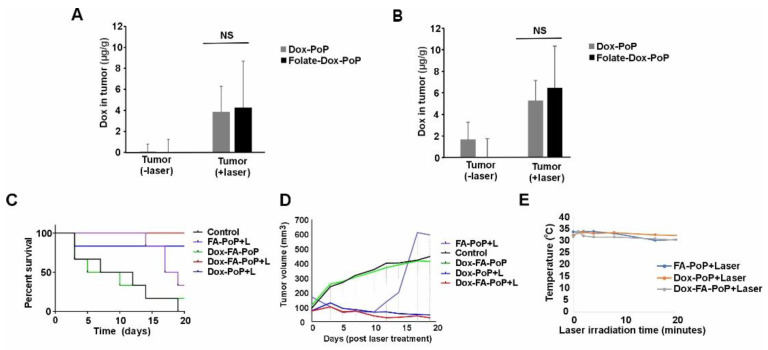
In vivo evalution of FA-PoP liposomes. Tissue distribution of Dox-PoP and Dox-FA-PoP liposomes injected in nude mice (4 mg kg^−1^ Dox) bearing dual KB tumors sacrificed after (**A**) 4 h, or (**B**) 24 h, *n* = 4 mice per group. An unpaired *T*-test was performed for laser-irradiated samples. NS indicated not significant by *t*-test. Tumors were treated with laser at 250 J cm^−2^ 1 h after i.v. injection via the tail vein. For tumor inhibition studies, nude mice bearing KB tumor were injected with 3 mg kg^−1^ and irradiated with a 665 nm laser at 400 J cm^−2^. (**C**) Kaplan–Meier survival curves for mice injected with indicated liposomes. (**D**) Tumor volume of nude mice injected with indicated liposomes. (**E**) Tumor surface temperature monitored during laser treatment.

Dox-PoP or Dox-FA-PoP liposomes at a dose of 4 mg kg^−1^ of Dox were injected in mice intravenously via the tail vein. Tissue Dox distribution was evaluated in nude mice bearing dual tumors. Post injection, tumors on the right flank were treated with a 665 nm laser at 150 J cm^−2^. Tumors on the left flank remained untreated and served as a control. Mice were sacrificed either after 4 h or 24 h, and key organs were harvested for Dox concentration analysis. Tumors without laser irradiation showed 4–6-fold lower Dox accumulation in comparison with Dox-FA-PoP treated with a laser (Figure 4A). As shown in previous studies, an increase in the accumulation of Dox post-laser irradiation may be attributed to vascular permeabilization owing to PDT effects in the treatment area [32,33,38]. Laser-treated tumors showed similar Dox concentration with Dox-PoP and Dox-FA-PoP, although Dox-FA-PoP was marginally higher. Tumors harvested at 24 h showed slightly higher Dox accumulation at 7 µg per gram compared to tumors excised after 4 h, which showed 4 µg per gram of tumor tissue (Figure 4B). These results indicate enhanced drug accumulation in the tumor due to tumor blood vessel permeabilization as a result of PDT.

Tumor inhibition studies were carried out in nude mice bearing single KB tumor xenografts. Mice were injected with Dox-PoP or Dox-FA-PoP liposomes at 3 mg kg^−1^ Dox or FA-PoP (empty) liposomes. Figure 4C shows the Kaplan–Meier survival curve for tumor inhibition study. Untreated control group showed a mean survival time of 9.5 days. The Dox-FA-PoP liposomes group which received laser treatement had the highest mean survival of 19 days. Empty FA-PoP liposomes treated with a laser had a margianlly lower mean survival time of 17.8 days. This result is in line with previous in vitro results where PDT had a major impact in tumor cell killing and Dox loading enhanced the tumor cell killing. Tumors treated with Dox-FA-PoP alone with no laser exposure exhibited mean survival time comparable to the control cohort. The untargeted Dox-PoP liposomes group treated with laser showed mean survival time 16.3 days. FA receptor targeting increased the survival of laser treated mice in comparison with untargeted Dox-loaded liposomes, but not significatly.

Figure 4D shows the tumor volume growth in mice treated as indicated. Folate liposomes and Dox-loaded FA liposome groups with no light exposure retarded tumor growth initially but did not sustain after 10 days post-laser treatment. On the other hand, Dox-PoP treated with a laser and Dox-FA-PoP treated with a laser sustained tumor growth inhibition. As observed in Kaplan–Meier survival curve results, folate receptor-targeted liposomes performed marginally better in hindering tumor growth in comparison with untargeted liposomes. Surface temperature of the tumor during laser treatement was monitored to discount the effects of photothermal therapy. As shown in Figure 4E, temeprature fluctuated between 28 and 34 °C leading to the inference that photothermal therapy was not a contributing factor. In vivo studies perfomed on nude mice bearing human A2780 intraperitneal tumors exhibited binding of FA lipsomes to tumor nodules around the kidneys revealing the potential of FA receptor-targeted PoP liposomes in imaging as well as a potential theranostic agent (Figure S1).

**Figure 5:**
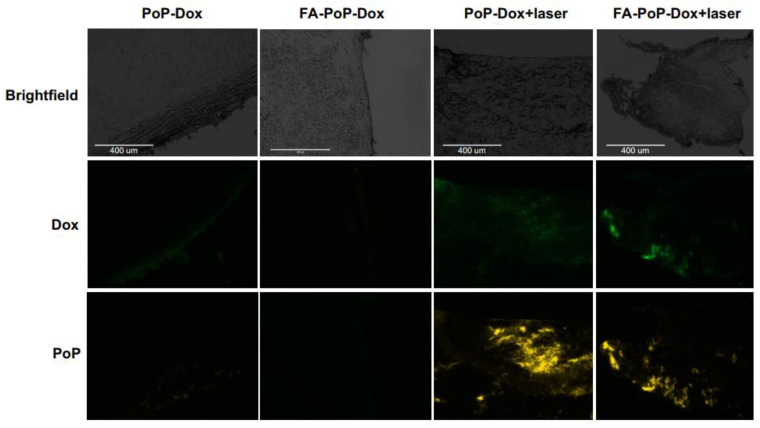
Fluorescence imaging of tumor slices obtained from KB tumor-bearing nude mice treated with Dox-PoP or Dox-FA-PoP with or without laser irradiation. Mice were sacrificed 4 h after injection.

Mice injected with Dox-PoP or Dox-FA-PoP were treated with a 665 nm laser and sacrificed after 4 h. Tumors were excised and snap frozen. Tumor slices prepared using a cryostat were imaged using a fluorescence microscope. Figure 5 shows the brightfield and fluorescence channels of Dox-PoP and Dox-FA-PoP tumor slices. The Dox channel (middle row) indicates small amounts of dox accumulating in tumor tissue with no light treatment. However, Dox-PoP + laser and Dox-FA-PoP + laser show higher Dox accumulation. An increase in dox accumulation in FA-positive Dox-loaded liposomes treated with light might be a result of vascular permeabilization due to laser irradiation. The intensity of Dox fluorescence in Dox-FA-PoP is higher than in Dox-PoP, as seen in Figure 5; however, no statistical analysis was carried out. As shown in the bottom row of Figure 5, PoP fluorescence data are in agreement with the Dox channel. Since Dox is loaded into PoP liposomes, with an increase in Dox signal, an increase in PoP fluorescence is expected if vascular permeabilization is driving the accumulation of liposomes in the tumor from circulation. More in-depth in vivo imaging studies are warranted to further understand the binding and uptake of PoP liposomes.

## 4. Discussion and Conclusions

The treatments presented here employed PDT in combination with chemotherapy for the treatment of cancer. However, the targeting and accumulation of drugs in the tumor region would benefit from improvement. Active targeted therapy, for example, folate receptor-targeting, aims for improved CPT efficacy. In this study, we examined the in vitro and in vivo efficacy of Dox-loaded photosensitive FA liposomes capable of selectively targeting folate receptors overexpressed in certain cancer cells. KB cells, which overexpress the FA receptor, had a higher uptake of targeted liposomes in vitro. In vivo, both targeted and untargeted liposomes had 4–6-fold higher Dox accumulation with light treatment. CPT using targeted and untargeted liposomes showed similar mean survival time. Therefore, it appears that FA-targeting did not improve tumor drug delivery in vivo, and rather, PDT was the major contributing factor in enhanced Dox accumulation. Further investigation into the efficacy and impact of chemotherapy in combination with PDT is warranted to improve the synergistic capabilities of FA receptor-targeted liposomes in vivo. The shorter half-life of FA liposomes might have resulted in rapid clearance of liposomes, which could have led to alternative therapeutic outcomes for targeted and untargeted cohorts treated with laser. The targeting strategy itself could be varied for in vivo applications, and other groups have reported liposomes with as low as 0.03% FA for FA receptor-targeting [39]. On the other hand, it has been shown in preclinical models that targeting antibodies does not improve overall drug biodistribution of liposomes, at least with intravenous administration [38]. The versatility of Dox-loaded PoP liposomes to be used as an intrinsic fluorescence imaging agent could facilitate theranostic approaches in future studies. Overall, the results from this study show that FA-targeted liposomal CPT is feasible and effective, but improvements in the in vivo targeting are required.

## Figures and Tables

**Table 1:** Size parameters of PoP and FA-PoP liposomes with and without Dox loading. Values show mean +/− std. dev. for triplicate measurements.

Liposomes	PoP	FA-PoP	PoP-Dox	FA-PoP-Dox
Diameter (nm)	102.06 ± 12.8	97.92 ± 2.0	100.97 ± 8.4	116.9 ± 0.8
Polydispersity index	0.102 ± 0.1	0.05 ± 0.03	0.081 ± 0.1	0.114 ± 0.01

## Data Availability

Data is available from the authors upon reasonable request.

## Supplementary Materials

The following supporting information can be downloaded at: https://www.mdpi.com/article/10.3390/pharmaceutics15102385/s1, Figure S1: In vivo imaging of nude mice bearing intraperitoneal A2780 tumor nodules injected with FA-PoP liposomes.
