# Supplementary material for: Folate-Targeted Nanoliposomal Chemophototherapy
Source: Pharmaceutics. 2023 Sep 26;15(10):2385. doi: 10.3390/pharmaceutics15102385 (PMC10609802; doi:10.3390/pharmaceutics15102385)
Supplement: Supplementary file 1 [file pharmaceutics-15-02385-s001.zip › pharmaceutics-2600105-supplementary.pdf]

Supporting Information: Folate-targeted Nanoliposomal Chemophototherapy

Upendra Chitgupi, Yiru Qin, Sanjana Ghosh, Breandan Quinn, Kevin Carter, Xuedan He and Jonathan F. Lovell

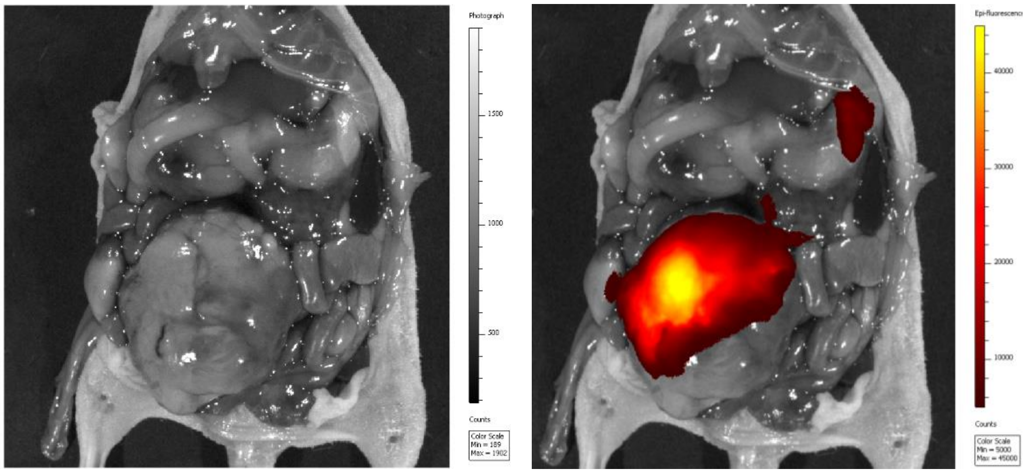

Figure S1. In vivo imaging of nude mice bearing intraperitoneal A2780 tumor nodules injected with FA-PoP liposomes and imaged following dissection.
